# Author Correction: Early stimulated immune responses predict clinical disease severity in hospitalized COVID-19 patients

**DOI:** 10.1038/s43856-023-00248-2

**Published:** 2023-02-02

**Authors:** Rebecka Svanberg, Cameron MacPherson, Adrian Zucco, Rudi Agius, Tereza Faitova, Michael Asger Andersen, Caspar da Cunha-Bang, Lars Klingen Gjærde, Maria Elizabeth Engel Møller, Patrick Terrence Brooks, Birgitte Lindegaard, Adin Sejdic, Zitta Barrella Harboe, Anne Ortved Gang, Ditte Stampe Hersby, Christian Brieghel, Susanne Dam Nielsen, Daria Podlekareva, Annemette Hald, Jakob Thaning Bay, Hanne Marquart, Jens Lundgren, Anne-Mette Lebech, Marie Helleberg, Carsten Utoft Niemann, Sisse Rye Ostrowski

**Affiliations:** 1 Department of Hematology, Copenhagen University Hospital, Rigshospitalet, Copenhagen, Denmark; 2 PERSIMUNE Center of Excellence, Copenhagen University Hospital, Rigshospitalet, Copenhagen, Denmark; 3 Department of Clinical Immunology, Copenhagen University Hospital, Rigshospitalet, Copenhagen, Denmark; 4 Department of Pulmonary and Infectious Diseases, Copenhagen University Hospital, Nordsjællands Hospital, Hillerød, Denmark; 5 Department of Clinical Medicine, University of Copenhagen, Copenhagen, Denmark; 6 Department of Virus & Microbiological Special Diagnostics, Division of Infectious Disease Preparedness and Research, Statens Serum Institut, Copenhagen, Denmark; 7 Department of Medicine, Section of Infectious Diseases, Herlev and Gentofte Hospital, Herlev, Denmark; 8 Department of Infectious Diseases, Copenhagen University Hospital, Rigshospitalet, Copenhagen, Denmark; 9 Department of Respiratory Medicine, Bispebjerg Hospital, Copenhagen, Denmark

**Keywords:** Viral infection, Innate immunity

Correction to: *Communications Medicine* 10.1038/s43856-022-00178-5, published online 12 September 2022.

An author, Zitta Barrella Harboe (Z.B.H.), had inadvertently been omitted from the author list of this article. This author has now been added and the author list is as follows: ‘Rebecka Svanberg, Cameron MacPherson, Adrian Zucco, Rudi Agius, Tereza Faitova, Michael Asger Andersen, Caspar da Cunha-Bang, Lars Klingen Gjærde, Maria Elizabeth Engel Møller, Patrick Terrence Brooks, Birgitte Lindegaard, Adin Sejdic, Zitta Barrella Harboe, Anne Ortved Gang, Ditte Stampe Hersby, Christian Brieghel, Susanne Dam Nielsen, Daria Podlekareva, Annemette Hald, Jakob Thaning Bay, Hanne Marquart, Jens Lundgren, Anne-Mette Lebech, Marie Helleberg, Carsten Utoft Niemann, Sisse Rye Ostrowski’.

Z.B.H. is affiliated with the Department of Pulmonary and Infectious Diseases, Copenhagen University Hospital, Nordsjællands Hospital, Hillerød, Denmark, and the Department of Clinical Medicine, University of Copenhagen, Copenhagen, Denmark.

The “Acknowledgements” section has been updated to include funding received by Z.B.H. and is now as follows: ‘The authors would like to acknowledge all the investigators and patients participating in this study. This work was supported by a grant from the Ministry of Higher Education and Science, Copenhagen, Denmark (0238-00006B) and was further supported by funding from the Lundbeck Foundation (R349-2020-835, received by Z.B.H.).’

The “Author contributions” section has been updated to include Z.B.H. and is now as follows: ‘S.R.O. and C.N. designed and initiated the study and share senior authorship. R.S., A.H., S.D.N. collected the data, and RS analyzed data and wrote the first draft of the manuscript. C.M. contributed with supervision and input for data analysis, discussion, and interpretation of results. A.Z., R.A., T.F., and L.K.G. gave input to data visuization, analysis, and interpretation. L.K.G., M.A.A., C.C.B., C.B., C.N. and S.R.O. contributed to the discussion and clinical interpretation of data and results. S.R.O. was responsible for the TruCulture analysis, and provided the TruCulture data. J.B. and H.M. analyzed and provided the flow cytometry data, and contributed to data interpretation. P.T.B., B.L., A.S., A.O.G., D.S.H., M.M., D.P., Z.B.H. and C.B. helped to include patients in the study and collect clinical data. J.L., A.M.L., and M.H. contributed to management of the study and to the discussion of the results. All authors participated in writing and editing the final manuscript.’

These changes have been made in the HTML and PDF versions of the article.

